# Profiling complex repeat expansions in *RFC1* in Parkinson’s disease

**DOI:** 10.1038/s41531-024-00723-0

**Published:** 2024-05-24

**Authors:** Pilar Alvarez Jerez, Kensuke Daida, Abigail Miano-Burkhardt, Hirotaka Iwaki, Laksh Malik, Guillaume Cogan, Mary B. Makarious, Roisin Sullivan, Jana Vandrovcova, Jinhui Ding, J. Raphael Gibbs, Androo Markham, Mike A. Nalls, Rupesh K. Kesharwani, Fritz J. Sedlazeck, Bradford Casey, John Hardy, Henry Houlden, Cornelis Blauwendraat, Andrew B. Singleton, Kimberley J. Billingsley

**Affiliations:** 1 Laboratory of Neurogenetics, National Institute on Aging, Bethesda, MD USA; 2 Center for Alzheimer’s and Related Dementias, National Institute on Aging, Bethesda, MD USA; 3 Department of Neurodegenerative Disease, UCL Queen Square Institute of Neurology, University College London, London, UK; 4 DataTecnica LLC, Washington, DC USA; 5 Sorbonne Université, Institut du Cerveau-Paris Brain Institute-ICM, Institut National de la Recherche Médicale-U1127, Centre National de la Recherche Scientifique, Paris, France; 6 UCL Movement Disorders Centre, University College London, London, UK; 7 Department of Neuromuscular Diseases, UCL Queen Square Institute of Neurology, London, UK; 8 Oxford Nanopore Technologies, Oxford, UK; 9 Human Genome Sequencing Center, Baylor College of Medicine, Houston, TX USA; 10 Department of Molecular and Human Genetics, Baylor College of Medicine, Houston, TX USA; 11 Department of Computer Science, Rice University, Houston, TX USA; 12 The Michael J. Fox Foundation for Parkinson’s Research, New York, NY USA

**Keywords:** Structural variation, DNA sequencing

## Abstract

A biallelic (AAGGG) expansion in the poly(A) tail of an AluSx3 transposable element within the gene *RFC1* is a frequent cause of cerebellar ataxia, neuropathy, vestibular areflexia syndrome (CANVAS), and more recently, has been reported as a rare cause of Parkinson’s disease (PD) in the Finnish population. Here, we investigate the prevalence of *RFC1* (AAGGG) expansions in PD patients of non-Finnish European ancestry in 1609 individuals from the Parkinson’s Progression Markers Initiative study. We identified four PD patients carrying the biallelic *RFC1* (AAGGG) expansion and did not identify any carriers in controls.

A biallelic pentanucleotide repeat expansion (AAGGG) in the poly(A) tail of an AluSx3 transposable element in the replication factor C subunit 1 (*RFC1*) gene is a frequent cause of cerebellar ataxia, neuropathy, and vestibular areflexia syndrome (CANVAS)^1^. Further, the length of the biallelic “AAGGG” expansion is disease-modifying, as an inverse correlation was observed between the size of expansions and age at neurological onset, age at onset of dysarthria and/or dysphagia, and age at the use of one stick^2^.

More recent genetic studies have broadened the phenotypic spectrum of *RFC1* expansions. Several groups have investigated the prevalence of *RFC1* expansions in Parkinsonian disorders including multiple system atrophy with conflicting findings^3,4^. In terms of its association with Parkinson’s disease (PD) specifically, Kyotovuori et al. identified that three out of 569 patients with PD were carriers for the biallelic *RFC1* (AAGGG) expansion, suggesting that this expansion may be a rare cause of PD in the Finnish population^5^.

In this study, we aimed to profile the biallelic *RFC1* “AAGGG” repeat expansion in PD patients from non-Finnish European ancestry in 903 cases and 706 controls from the PPMI cohort. Due to the complexity of the *RFC1* repeat, short-read whole genome sequencing (WGS) data can yield false positives, hence experimental validation is required. From the short-read analysis, five patients were predicted to carry the biallelic expansion. However, through the Oxford Nanopore Technologies (ONT) long-read DNA WGS, one predicted carrier was identified as a false positive, leaving four validated carriers. The four remaining carriers were PD patients resulting in an estimated frequency of 0.43% in PD. No controls carried the “AAGGG” *RFC1* repeat expansion. From the ONT long-read analysis, the biallelic “AAGGG” expansion repeat units varied from 333 to 1183 in the four carriers, which is slightly larger than the 144–820 reported in PD patients in the Finnish population^5^, but a smaller range than what was observed in CANVAS patients from European ancestry, which ranged from 400 to 2000 repeats^1^ (Supplementary Table 2).

For the four “AAGGG” RFC1 carriers, some variation was observed in the clinical phenotypic description (Table 1). However, overall in agreement with previous observations of the repeat expansion in PD patients, the clinical phenotype was that neither the presentation nor disease course differed from those in other PD patients. Patient 1 developed PD at the age of 57. She presented tremors, rigidity, and bradykinesia as motor symptoms (MDS-UPDRS 24 pts, Hoen and Yahr (H&Y) stage 2), depression, mild cognitive decline, constipation, and insomnia as non-motor symptoms. Her symptoms did not show much progression until the latest PPMI visit (one and a half years after onset) since she had not taken any medications. Her dopamine transporter (DaT) imaging was normal at the initial diagnosis and she showed a negative reaction in alpha-synuclein (aSyn) SAA.

**Table 1:** Clinical characteristics of the four PD patients “AAGGG” biallelic *RFC1* expansion carriers

ID	Patient 1		Patient 2		Patient 3		Patient 4	
Diagnosis	PD		PD		PD		PD	
SAA	0		0		1		_	
GBA1	_		_		_		_	
LRRK2	_		_		_		G2019S	
SEX	Female		Female		Female		0	
FAMILY_HISTORY	1		0		0		1	
Age at baseline	57.7		65.8		54.7		77.3	
Age at onset	57.7		65.7		54.5		76	
UPSIT	33		34		20		37	
UPSIT percentile	20.5		50		1		NA	
Hyposmia	0		0		1		NA	
RBD	0		1		0		0	
Depression	1		1		0		0	
MCI	1		0		0		0	
Constipation	1		1		0		1	
Daytime sleepiness	0		0		0		0	
Insomnia	1		1		0		1	
Mean_caudate	3.72		2.93		2.43		NA	
Mean_putamen	2.54		1.92		1.09		NA	
Mean_striatum	3.13		2.42		1.76		NA	
Follow-up years		1.51		1.91		7		_
Levodopa dosage	0	0	0	600	0	450	_	_
LEDD	0	0	0	900	0	900	_	_
Hoen and Yahr stage	2	2	1	3	1	2	2	_
Tremor	1		1		1		NA	
Rigidity	1		1		0		NA	
Bradykinesia	1		1		1		NA	
Postural instability	0		0		0		NA	
MDS-UPDRS 1	9	10	24	19	2	8	5	_
MDS-UPDRS 2	5	3	17	27	2	6	1	_
MDS-UPDRS 3	24	22	18	35	14	7	16	_
MDS-UPDRS 4	0	0	0	0	0	0	0	_
MOCA	23	_	29	28	29	30	25	_

*SAA* seeding amplification assay, *UPSIT* the University of Pennsylvania Smell Identification Test, *RBD* rapid eye movement sleep behavior disorder, *MCI* mild cognitive impairment, *LEDD* levodopa equivalent daily dose, *MDS-UPDRS* Movement Disorder Society-sponsored revision of the unified Parkinson’s disease rating scale, *MOCAMo* Montreal cognitive assessment.

Patient 2 developed PD at the age of 65. She presented tremors, rigidity, bradykinesia, and postural instability at the diagnosis (H&Y stage 1, MDS-UPDRS part 3, 18 points). Approximately 2 years after the onset, her symptoms progressed (H&Y stage 3, MDS-UPDRS part 3, 35 points) with 900 mg of levodopa equivalent dose (LEDD) and she showed a negative reaction in aSyn SAA.

Patient 3 developed PD at the age of 54, presenting with tremors, bradykinesia, and hyposmia. At the age of 61, 9 years from the onset, her H&Y stage was 2 with 900 mg of LEDD. She showed a positive reaction in aSyn SAA. DaT imaging showed decreased binding in the putamen.

Patient 4 developed PD at the age of 76. A year after the onset, his H&Y stage was 2, MDS-UPDRS part 3 was 16, accompanied by constipation and insomnia. Clinical data of follow-up visits were not available and aSyn SAA was not performed for this patient. Genetic testing revealed that he was a carrier of the known damaging *LRRK2* p.G2019S variant. DaT imaging results were not available.

In this study, we leveraged short-read WGS data from the PPMI cohort and the computational tool str-analysis to genetically screen 1609 individuals for the biallelic “AAGGG” repeat expansion and identified four PD patient carriers and no control carriers giving a frequency of 0.44% in PD. To note, when we excluded carriers of known pathogenic variants in *LRRK2*, *GBA1*, and *SNCA*, and those with scans without evidence of dopaminergic deficits (SWEDD), the estimated frequency in PD is higher (0.84%), which is slightly higher than what was previously reported in the Finnish population, who report a frequency of 0.53% in PD patients^5^.

Interestingly, the reported clinical phenotype of these patients is in line with typical PD symptoms and no clear red flags in the clinical data were observed that the diagnosis was incorrect. However, it is worth noting that no specific ataxia phenotype data is collected and therefore we cannot exclude misdiagnosis. Actually, only one out of three patients that had data available showed a positive reaction in aSyn SAA. Notably, SAA positivity is generally very high in PPMI PD cases and is influenced by genetic status. 67.5% of LRRK2 cases are SAA positive, whereas typical non-LRRK2 cases show a remarkably high SAA positivity rate of 93.3%.

As demonstrated in this study, long-read DNA sequencing is a powerful tool and a required step to validate potential pathogenic repeat expansion carriers. Short-read sequencing methods are notorious for over or underestimating repeat expansion lengths and the RFC1 locus is further complicated by its variable motif sequence. Therefore, although the allele frequency reported in this present study is inline if not slightly higher than what was identified in the Finnish study using PCRs for large (XL-PCR) amplicons and repeat primed PCR (see ref. ^5^), given the limitations, short-read sequencing can still lead to false negatives. As such, generating population-scale long-read DNA sequencing datasets to capture repeat expansions that are currently hidden using traditional methods is an essential step towards solving the architecture of complex genetic disorders^6^. For PD specifically, the Global Parkinson’s Genetics Program (GP2 www.gp2.org) is leading a large-scale initiative to long-read DNA sequence ~1000 case-control samples (Fig. 1)

**Fig. 1:**
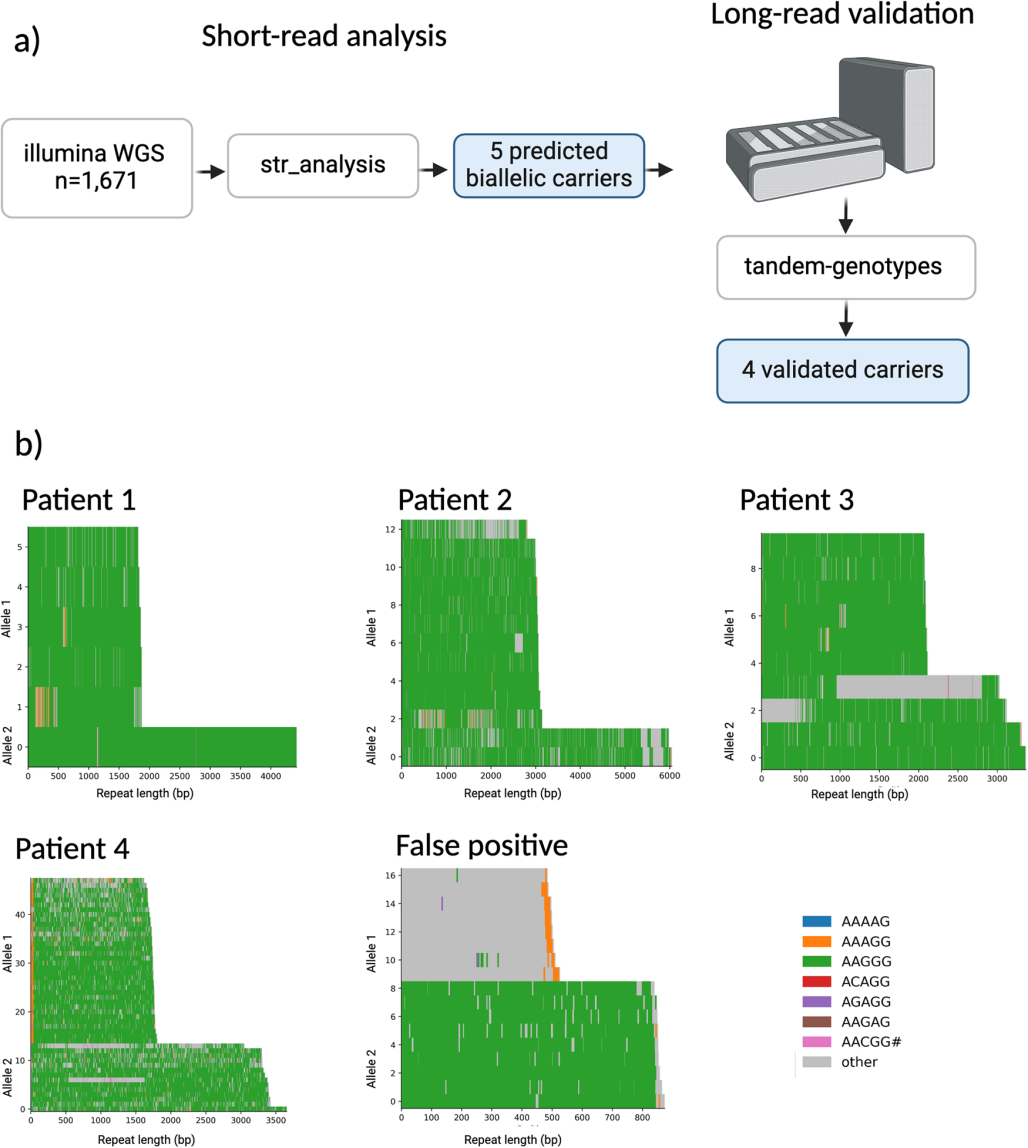
Investigation of the RFC1 biallelic expansion in PD patients from European ancestry. **a** Overview of the study design and rationale behind the analysis included in the work. **b** Waterfall plots of the ONT long-read sequencing data showing the four predicted carriers and one false positive. Created with BioRender.com.

## Methods

### Cohort information

Samples were obtained from the Parkinson’s Progression Markers Initiative (PPMI; https://www.ppmi-info.org/). Clinical and demographic characteristics of the PPMI cohort are shown in (Supplementary Table 1). Participants included PD cases clinically diagnosed by experienced neurologists and control individuals. All PD cases met the criteria defined by the UK PD Society Brain Bank^7^. All individuals were of European descent and were not age or gender-matched. This included a total of 903 cases and 706 neurologically healthy controls. PD cases ranged from 33 years to 90 years of age at diagnosis (mean 61.7 ± 11.05, median 63.0) and included 62 individuals who showed SWEDD and 368 individuals who carry known genetic mutations associated with PD (within *LRRK2*, *GBA1,* and *SNCA*). Control subjects ranged from 19 years to 86 years of age (mean 58.3 ± 11.54, median 60.0) and included 503 individuals who carry known genetic mutations associated with PD.x.

#### Short-read analysis

Short-read whole-genome sequencing data in bam format was downloaded through AMP-PD and has been reported in detail previously by Iwaki et al. ^8^. For short-read data analysis, alignment was performed based on the GATK best practice pipeline, and the fastqs were aligned to the hg38 reference genome using BWA-mem. The STR detection tool str-analysis was used to screen for biallelic *RFC1* (AAGGG) expansion carriers (https://github.com/broadinstitute/str-analysis).

### Long-read validation of expansion in carriers

To validate the five individuals predicted to carry pathogenic *RFC1* “AAGGG” biallelic repeat expansions, ONT whole-genome long-read DNA sequencing was performed. For all predicted carriers, a library was prepared from the DNA of the individuals with either the SQK-LSK110^9^ or the SQK-LSK114 ligation sequencing kit from ONT^10^. The samples were quantified using a Qubit fluorometer and were loaded onto a PromethION R.9.4.1 (SQK-LSK110) or R.10.4 flow cell (SQK-LSK114) following ONT standard operating procedures and ran for a total of 72 h on a PromethION device (Supplementary Table 2).

Fast5 files containing the raw signal data were obtained from sequencing performed using MinKNOW (ONT). All fast5 files were used to perform super accuracy base calling on each sample with Guppy v6.0.1 (R.9) (ONT) or Dorado (v0.5.0). and sequencing statistics were obtained with seqkit v2.2.0 using fastq files that passed quality control filters in the super accuracy base calling. To accurately determine the length of *RFC1* repeat expansion from the ONT data, as required by tandem-genotypes, the fastqs were first mapped to the hg38 reference using LAST as described in detail here (https://github.com/mcfrith/last-rna/blob/master/last-long-reads.md)^11^. To size the expansion on each allele, tandem genotypes were then run using the mapped files^12^.

### Reporting summary

Further information on research design is available in the Nature Research Reporting Summary linked to this article.

### Supplementary information

Supplementary Tables

Related Manuscript File

## Data Availability

Data used in the preparation of this article were obtained from the PPMI database (www.ppmi-info.org/access-data-specimens/download-data), RRID: SCR_006431. For up-to-date information on the study, visit www.ppmi-info.org. The PPMI cohort and the ONT raw data will be available at the LONI IDA.
